# Dehorning Cattle

**Published:** 1890-09

**Authors:** 


### CASES, SELECTIONS.

### DEHORNING CATTLE.[1]

THE solution of the question as to whether it is cruel to perform any operation of a painful nature upon animals must be based upon the necessity for the operation. The infliction of unnecessary pain is cruelty, and, therefore, if a person performs an unnecessary operation which entails suffering, he is guilty of cruelty, and any one instigating thereto must also be held guilty as an accessory. The *jus animalium* demands that this view be adopted, and it is recognized in courts of justice as the law of the land. As to what operations are necessary, and what are cruel, because not needed, we will not venture to discuss: but it may be observed that even among veterinary experts there is a wide difference of opinion on this point. Certainly, there are operations which must be performed on healthy animals—putting aside altogether those for the cure, prevention of, or relief from disease or deformity—though even in the performance of these there may be cruelty if they are unskillfully or barbarously conducted. Operations which render animals more useful to man, or without

[1] *The Veterinary Journal*, August, 1890.

which they perhaps could not be safely utilized by him—castration of the horse, for instance—cannot be regarded as cruel, though humanity requires that they be performed with skill and as little pain as possible; but those undertaken with the object of *improving nature*, and which may be more fitly termed mutilations designed to gratify a depraved taste or stupid fashion—such as "cropping" dogs' ears and tails, "dubbing" fowls, and "docking" all riding horses' (and, it might be asserted, 99 per cent. of harness horses') tails—should be classed as cruel, and therefore a violation of the law. Not only do they occasion pain, and often immediate grave results, but they cause serious inconvenience and discomfort to the animals afterward—as, for instance, the torture docked animals experience from flies.

The practice of dehorning or dishorning cattle has revealed the usual divergence of veterinary opinion as to what is cruelty and what is not cruelty, and has given judges and the public another opportunity of commenting upon the unreliability of professional knowledge. The testimony given by veterinary witnesses has fairly baffled those before whom test cases have been brought for judgment, and has necessitated appeals to the highest courts of law, where common sense may prevail in settling the question, apart altogether from the vagaries of scientific minds. It has been decided by the High Court of Justice that in England dehorning is cruel, and therefore unlawful; but in Scotland and Ireland no final decision has yet been arrived at. It will be a curious result of the pending appeals in these countries if what in England is declared to be illegal is pronounced perfectly lawful in them.

That dehorning is painful there should be no doubt whatever in the mind of any one who knows the anatomy of the parts operated, even when the operation is performed by a skillful operator; but when it is done by a clumsy amateur with imperfect instruments, then, of course, the pain becomes increased, and amounts to torture. And not only is there the immediate pain of sawing through the sensitive structures and the bone which forms the core of the horn, but the after-effects lead to suffering, and sometimes a serious termination is observed. The hemorrhage is sometimes so great that the wound has to be cauterized. Inflammation of the mucous membrane lining the sinuses of the head is a frequent occurrence, leading to suppuration, and in some instances to gangrene, which usually ends in death. Not unfrequently, when the inflammation in the sinuses is severe, a more or less violent form of ophthalmia in one or both eyes ensues. In some cases the wound does not heal completely, and fistulæ appear, which give exit to pus, and Lafosse has reported inflammation of the brain as a sequel of amputation of the horns.

But if it be conceded that the operation is necessary in the interests of cattle dealers, and also in that of the animals themselves—a matter which is disputed—it must be admitted that it should be conducted skillfully and properly, so as to entail as little suffering as possible; and to secure this most desirable object, surely an anæsthetic might be employed. In fact, the operation is one that should certainly be undertaken by a veterinary surgeon, not by a farm laborer.

It is a question, however, whether the operation—granting always that it is necessary—does not amount to cruelty when performed after a certain age. In early life (at 2 or 3 months old) the rudimentary horns can be completely extirpated (as shown and described in Fleming's "Text-book of Operative Veterinary Surgery," Part I, pp. 255, 256) with no danger and very little pain. This is the time when operative procedure ought to be resorted to, and it should be considered a legal offence to subject cattle to the torture of amputation of the horns when, through carelessness or apathy, these organs have been allowed to become fully developed. No excuse should be allowed to prevail under such circumstances, and we are certain that no veterinary surgeon who values the reputation of his profession with regard to humanity will be found to offer any. If oxen must be bred with horns which are a source of inconvenience and danger in adult life, then these appendages should be removed at a suitable time, when such removal is very easy and entails but little suffering and no danger to the animal. Such a course should surely commend itself to cattle breeders as far more humane and business-like than the stupid and brutal practice which now prevails, and which, while it gives lawyers employment, fills with disgust those who have any feeling for animals, at the same time displaying the hopelessly divergent opinions of veterinary experts.

